# Deficiencies in immunoassay methods used to monitor serum Estradiol levels during aromatase inhibitor treatment in postmenopausal breast cancer patients

**DOI:** 10.1186/2193-1801-2-5

**Published:** 2013-01-11

**Authors:** Jenny Jaque, Heather Macdonald, Doerthe Brueggmann, Sherfaraz K Patel, Colleen Azen, Nigel Clarke, Frank Z Stanczyk

**Affiliations:** Department of Obstetrics and Gynecology, Keck School of Medicine of the University of Southern California, Los Angeles, CA USA; Southern California Clinical and Translational Science Institute, University of Southern California Keck School of Medicine, Los Angeles, CA USA; Steroids Department, Quest Diagnostics Nichols Institute, San Juan Capistrano, CA USA; Department of Preventive Medicine, Keck School of Medicine of the University of Southern California, 90089 Los Angeles, CA USA

## Abstract

Optimal care for breast cancer patients undergoing aromatase inhibitor (AI) treatment is ensured when estradiol (E_2_) levels are adequately suppressed. To assess treatment efficacy accurately, it is important to measure the serum E_2_ levels using a well validated assay method with high sensitivity and specificity. This translates into the urgent need to evaluate various E_2_ immunoassay kits, which are frequently used in hospital settings to measure E_2_ serum levels in patients undergoing AI treatment, so clinicians obtain accurate and reliable measurements allowing appropriate clinical decision making. Our objective was to evaluate the performance of different commercially available and commonly used E_2_ immunoassay kits regarding measurement of E_2_ levels in the serum of postmenopausal breast cancer patients treated with AIs, in comparison to a highly accurate and reliable mass spectrometry assay. Clinical and demographic data were obtained from 77 postmenopausal breast cancer patients who were treated with an AI. Serum E_2_ levels were measured by 6 immunoassay methods and by liquid chromatography-tandem mass spectrometry (LC-MS/MS), which served as the standard for comparison. Analysis of E_2_ by LC-MS/MS showed that 70% of the samples had levels that were <5 pg/ml. Three of the assays carried out with commercial E_2_ immunoassay kits had poor sensitivities and were not able to detect E_2_ levels <10 or <20 pg/ml. Although two of the E_2_ assays using commercial kits demonstrated a better sensitivity (5 pg/ml), the measured E_2_ values were substantially higher than those obtained by LC-MS/MS. The assay with the sixth commercial E2 kit grossly underestimated the true E_2_ values. E_2_ assays carried out with commercial E_2_ immunoassay kits lack the accuracy to measure the very low serum E_2_ levels found in patients being treated with AIs. Serum samples from such patients should be sent to laboratories that use a mass spectrometry assay.

**Electronic supplementary material:**

The online version of this article (doi:10.1186/2193-1801-2-5) contains supplementary material, which is available to authorized users.

## Introduction

Hormone suppression in postmenopausal women with estrogen or progesterone receptor positive breast cancers has been associated with significant benefits such as decreased local and distant recurrences, a lower risk of contralateral breast cancer and improved breast cancer specific mortality (Coates et al. 2007; Forbes et al. 2008). In patients with early breast cancer, adjuvant therapy with aromatase inhibitors (AIs) was proven to lower 5-year relapse rates with improved side effect profiles over alternative medications such as tamoxifen (Howell et al. 2005). Even women with metastatic disease benefit from slowed disease progression due to AI treatment. Therefore, AIs play a major role in the adjuvant treatment of estrogen receptor positive breasts cancers in postmenopausal patients.

In postmenopausal women, the primary source of estrogen is adipose tissue. Here, the enzyme aromatase converts testosterone and androstenedione into estradiol (E_2_) and the weaker estrogen, estrone, respectively. Third generation AIs suppress aromatase activity by 90 – 99%, which leads to a reduction of circulating estrogen levels to 1% to 10% compared with pretreatment levels (Santen et al. 2007). By decreasing systemic estrogen significantly, AIs prevent growth of estrogen receptor positive micrometastases and dormant cancer cells (Howell et al. 2005). Hence, full benefits and optimal treatment outcome of postmenopausal breast cancer patients depend on maximally suppressed E_2_ levels.

During AI treatment, mean (± standard deviation) serum levels of E_2_ have been reported to be 5.8 ± 4.1 pg/ml, as measured by radioimmunoassay (RIA) with preceding purification steps (Santen et al. 2007). To assess treatment efficacy correctly, it is important to measure these low E_2_ levels using an accurate and reliable assay method with high sensitivity and specificity such as gas or liquid chromatography-tandem mass spectrometry (GC-MS/MS or LC-MS/MS). Although being the purported “gold standard” for measuring low serum levels of E_2_, mass spectrometry is only available in a relatively small number of clinical diagnostic laboratories because it is a highly specialized and an expensive assay method (Stanczyk & Clarke 2010).

In most hospital settings, E_2_ measurements are obtained by a direct immunoassay technique. Using kits provided by various manufacturers, direct immunoassays are easy to carry out, allow automated performance and require only a small sample volume. However, direct immunoassays lack a purification step to remove metabolites that may potentially cross-react with the antibody in the assay (Stanczyk et al. 2010; Stanczyk et al. 2003). Hence, E_2_ levels in serum from patients treated with AIs may be measured significantly higher by direct immunoassay than by a mass spectrometry assay. These incorrectly elevated results can be attributed to the lower specificity of the direct immunoassay compared to mass spectrometry.

Thus, when comparing the specificity of a mass spectrometry assay versus a direct immunoassay, E_2_ levels in serum from patients treated with AIs may be measured significantly higher by direct immunoassay than by a mass spectrometry assay. Hence, direct immunoassays may yield incorrectly elevated results.

Another limitation of direct E_2_ immunoassays using commercial kits is that only a single small aliquot (0.1 ml) of serum can be used in the assay, as based on the procedure established by the kit manufacturer. Larger serum aliquots would compensate for a less sensitive E_2_ assay when very low E_2_ levels are being measured.

Inaccurate measurements of systemic E_2_ levels in a patient undergoing AI treatment may falsely indicate that the treatment goal is not reached, which can lead to a change in therapy. Subsequently, serious side effects could result such as rapid bone density loss or cardiovascular events in women with preexisting heart disease (Amir et al. 2011). In order to address this clinical need for correct E_2_ measurements, the objective of the present study was to evaluate the accuracy of several different commercially available and commonly used E_2_ immunoassay kits regarding measurement of E_2_ levels in the serum of postmenopausal breast cancer patients treated with AIs.

## Materials and methods

### Subjects

Study participants were naturally or surgically postmenopausal women, who had a diagnosis of breast cancer verified by histology. Seventy-seven patients were identified at the Medical Oncology service at the Los Angeles County and University of Southern California Medical Center (Los Angeles, CA), and were being treated with an aromatase inhibitor, which included either Arimidex (N = 63), Letrozole (N = 7), Femara (N = 4), or Aromasin (N = 3). The ages of the participants ranged from 33 to 79 years, and their BMI ranged from 16.2 to 49.4 kg/m^2^.

This study was approved by the Institutional Review Boards at USC.

### Blood sampling

A single blood sample (10 ml) was obtained from each subject and allowed to clot for 1–2 hours. After centrifugation of the sample, the serum was removed and stored at −20°C.

### Assay methods

#### Reference assay

The E_2_ LC-MS/MS assay was carried out at Quest Diagnostics Nichols Institute (San Juan Capistrano, CA). Each sample is prepared by adding 300 μL of 30% aqueous ethanol solution and 50 μL of the internal standard solution (which consists of the deuterated estradiol in methanol) to 200 μL of the serum. The ethanol solution is used to break up the ionic interaction from the carrier protein to release the analyte without precipitating. After vigorous mixing, the samples are incubated at room temperature for 15 to 20 minutes prior to being placed in the refrigerated autosampler for injection into the Aria TLX System (Thermo Fisher, San Jose, CA). Following injection, the sample is loaded onto a high flow rate extraction column. This creates turbulence inside the column, which allows the steroids to bind to the large particles of the extraction column, while protein and other debris freely flow through and are discarded. Following the loading step, the flow is reversed and the sample is eluted off the extraction column and transferred to a reverse-phase ether-linked phenyl analytical column. A binary HPLC gradient is applied to the column resulting in the separation of estradiol from its metabolites.

E_2_ is then quantitated using a TSQ Quantum Ultra triple quadrupole tandem mass spectrometer (Thermo Fisher, San Jose, CA). The tandem mass spectrometer permits the isolation of the parent compound within ± 0.5 m/z in the first quadrupole (Q1). In the second quadrupole (Q2), the parent ions collide with an inert gas (argon) to generate daughter ions, which are selected in the third quadrupole (Q3).

The sensitivity of the LC-MS/MS assay is 2 pg/ml; the intraassay coefficients of variation (CVs) are 15.3%, 10.4% and 7.6% at 10 pg/ml, 200 pg/ml and 800 pg/ml, respectively; the interassay CVs are 7.7-15.3%, 9.9-14.0% and 4.2-10.5% at 10 pg/ml, 200 pg/ml and 800 pg/ml, respectively.

#### Evaluated assays

Six different commercial immunoassay kits for measuring E_2_ were evaluated. The source and type of assays obtained with each of the kits are specified in Table 1. Four of the E_2_ kits (Beckman Coulter Ultra-Sensitive, Siemens Healthcare Diagnostics Coat-A-Count and Double Antibody, and Pantex Extraction^125^I) required radioimmunoassay (RIA) methodology, which was carried out manually, and the other two (Siemens Healthcare Diagnostics Immulite 2000 and Roche Diagnostics Elecsys 2010) required chemiluminescent immunoassay methodology using an automated analyzer. Only the assay using reagents from the Pantex Extraction^125^I kit required a preceding purification step.

**Table 1:** **Source and description of commercial E**_**2**_ **kits used to measure E**_**2**_ **following aromatase inhibitor treatment**

Company	Kit	Type of Assay	Sensitivity reported by manufacturer	Lowest E_2_Concentration on Standard Curve in our study*
Siemens Healthcare Diagnostics (Plainfield, IL)	Immulite 2000 Estradiol	Automated Direct Chemiluminescent Immunoassay	20	20
Siemens Healthcare Diagnostics (Plainfield, IL)	Coat-A-Count Estradiol RIA	Manual Direct RIA on coated Tube	8	20
Siemens Healthcare Diagnostics (Plainfield, IL)	Double Antibody Estradiol RIA	Manual Direct RIA	1.4	5
Beckman Coulter (Brea, CA)	Ultra-Sensitive Estradiol RIA	Manual Direct RIA	2.2	5
Roche Diagnostics (Indianapolis, IN)	Elecsys 2010 Estradiol II	Automated Direct Electro-chemiluminescent Immunoassay	5	5
Pantex Santa Monica, CA)	Extraction ^125^I Estradiol RIA	RIA with preceding extraction Step	10	10

* represents sensitivity of the evaluated assay.

Validation of the E_2_ assays with the 6 different kits, as based on the assay performance characteristics described in the package insert accompanying each kit, can be summarized as follows. Assay precision and linearity are acceptable. To validate assay specificity only a limited number of E_2_ metabolites were evaluated for cross-reactivity, and no comparison was made to an E_2_ immunoassay with preceding organic solvent extraction and chromatography steps. The assay sensitivity for each kit is shown in Table 1, but no information is given about how the assay sensitivity was determined. Finally, assay accuracy was not determined by comparing E_2_ values obtained with the kits to corresponding values obtained with a mass spectrometry assay.

## Results

### LC-MS/MS reference assay

**Figure 1:**
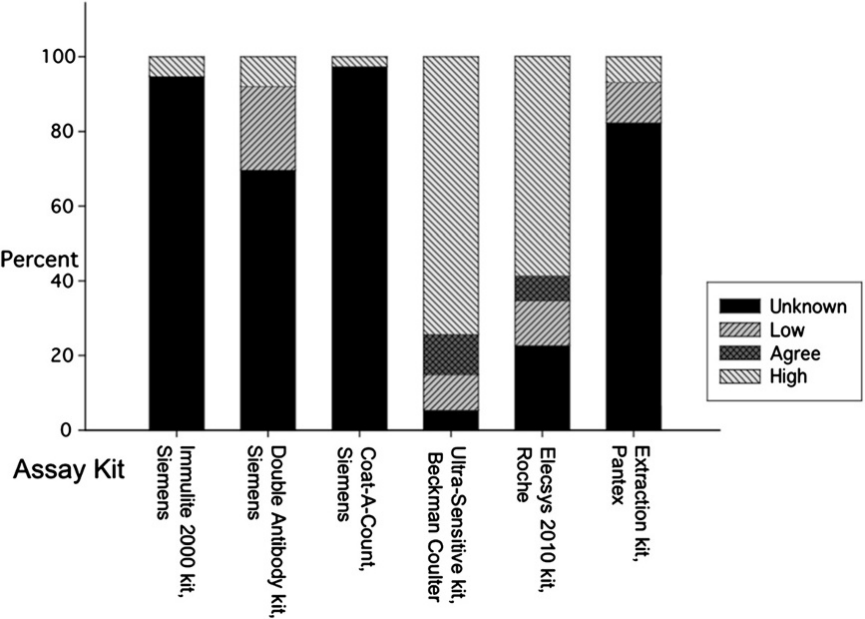
**Percent agreement of E**_**2**_**values obtained by the 6 different immunoassay methods with the corresponding values measured by LC-MS/MS.** Each bar graph shows the proportion of E_2_ values that are unknown (could not be compared to the LC-MS/MS values due to insufficient assay sensitivity), lower, in agreement, and/or higher than the values obtained by the mass spectrometry assay. Percent agreement of E_2_ values obtained by different immunoassay methods with the corresponding values measured by LC-MS/MS.

All 77 samples were quantified by LC-MS/MS assay. Of the 76 E_2_ measurements obtained by LC-MS/MS, 46 were below the sensitivity of the assay, which is 2 pg/ml. Another 10 values ranged between <2-5 pg/ml. Thus, approximately 73% of the values were <5 pg/ml. The remaining values ranged between 5–20 pg/ml. Figure 1 displays the degree of agreement between LC-MS/MS levels and the other 6 assay methods.

One of the E_2_ values measured by LC-MS/MS was very high (133 pg/ml) and clearly an outlier, most likely due to the patient not taking the aromatase inhibitor drug on the blood-sampling day. Consequently, the E_2_ results for that sample are excluded from the results of each of the assay methods.

### Kits from Siemens Healthcare Diagnostics

Three of the E_2_ assay methods involved use of reagent kits from Siemens Healthcare Diagnostics.

### Siemens Healthcare Diagnostics Immulite 2000 Kit

The assay carried out by chemiluminescent immunoassay using the Immulite 2000 kit has a sensitivity of 20 pg/ml according to the manufacturer, which is the same as the lowest point on the standard curve. A total of 72 out of 76 samples had E_2_ levels that were <20 pg/ml, and therefore no actual values below 20 pg/ml could be reported for the 72 samples (Figure 1, column 1). Four samples that were below 6 pg/ml by LC-MS/MS had levels between 21 and 44 pg/ml.

### Siemens Healthcare Diagnostics Double Antibody RIA Kit

The E_2_ assay with the Siemens Double Antibody RIA kit has a sensitivity of 1.4 pg/ml according to the kit manufacturer, but the lowest E_2_ point on the standard curve has a concentration of 5 pg/ml. A total of 70 of the 76 measurements obtained with this assay were <5 pg/ml, including 17 values that were above 6 pg/ml with LC-MS/MS (Figure 1, column 2). Two of the E_2_ values were very high (242 and 316 pg/ml) and are most likely due to cross-reactivity of the antibody with interfering substances in the serum samples. Four other samples had E_2_ values exceeding LC-MS/MS levels by 4 – 58 pg/ml.

### Siemens Healthcare Diagnostics Coat-A-Count RIA Kit

The sensitivity of the E_2_ assay with the Siemens Coat-A-Count RIA kit is 8 pg/ml according to the kit manufacturer, but the lowest point on the standard curve has a concentration of 20 pg/ml. Of the 76 E_2_ measurements with this assay, actual values for 74 of the samples that were below 20 pg could not be reported (Figure 1, column 3). Two of the values (41 and 86 pg/ml) were above 20 pg/ml, although the corresponding LC-MS/MS levels were <5 pg/ml.

### Beckman Coulter ultra-sensitive Estradiol RIA Kit

The E_2_ assay with this kit has a sensitivity of 2.2 pg/ml according to the kit manufacturer, but the lowest E_2_ standard on the standard curve is 5 pg/ml. Four of the E_2_ values with this assay were <5 pg/ml, so the actual values are unknown. However, of the 70 samples that had E_2_ values >5 pg/ml, 8 of the values agreed with and 7 were lower compared to the corresponding LC-MS/MS levels (Figure 1, column 4). A total of 55 values exceeded the corresponding LC-MS/MS levels by 3–13 pg/ml.

### Roche Diagnostics Elecsys 2010 Estradiol II Chemiluminescent Immunoassay Kit

This E_2_ assay, which is carried out on an analyzer, has a sensitivity of 5 pg/ml, which is the same as the lowest E_2_ standard on the standard curve. Of the 75 E_2_ measurements, 5 values were in agreement, and 9 values were lower, 44 values were higher, and 17 are unknown compared to the corresponding LC-MS/MS levels (Figure 1, column 5).

### Pantex Extraction ^125^I Estradiol RIA Kit

The E_2_ assay carried out with this kit was the only one that used a preceding purification step (organic solvent extraction). The sensitivity of this assay is 10 pg/ml, which is the same as that of the lowest E_2_ standard used in the standard curve. Of the 74 E_2_ measurements, 62 were <10 pg/ml and the values for these samples could not be reported. Seven values were below the corresponding LC-MS/MS levels, which ranged from 11 to 20 pg/ml, and 5 values were from 8–12 pg/ml higher than the corresponding LC-MS/MS levels (Figure 1, column 6).

## Discussion

The goal of this study was to evaluate the performance of commercially available E_2_ immunoassay kits commonly used to measure E_2_ levels in the serum of postmenopausal breast cancer patients treated with AIs. Our results clearly demonstrate that the investigated kits lacked the sensitivity and accuracy to detect the extremely low E_2_ levels in our patient group.

Optimal adjuvant therapy for early hormone sensitive breast cancer patients includes estrogen deprivation, either by blocking estrogen receptors at the target tissues using selective estrogen receptor modulators (SERMs), e.g., tamoxifen, or by decreasing estrogen production through inhibition of aromatase activity using AIs. In hormone sensitive breast cancers, hormone manipulation improves 15-year breast cancer mortality. Meta-analyses of randomized controlled trials of AIs versus tamoxifen in postmenopausal women with early hormone sensitive breast cancer demonstrated decreased distant recurrence, and improved cancer related mortality, disease free survival and event free survival (Coates et al. 2007; Forbes et al. 2008). Overall survival was better in the AI group as well (RR 0.71; p = 0.04). Additionally safety data from the Arimidex, Tamoxifen, Alone, or in Combination (ATAC) trial that compared the AI, anastrozole, with tamoxifen showed fewer treatment-related adverse events in the anastrozole treated group (Howell et al. 2005).

In perimenopausal women undergoing hormonal manipulation for early breast cancer, sequential use of tamoxifen and AIs has been shown to decrease recurrence risk over tamoxifen alone by 40% (Dowsett et al. 2010). Thus, perimenopausal women with low serum estrogen levels benefit more from aromatase inhibition than hormone receptor blockade with tamoxifen, when used either sequentially with tamoxifen or AIs alone. Accurate measurements of serum E_2_ levels are key to identifying which therapy most benefits women in this subgroup.

Lack of compliance, altered pharmacokinetics of drugs, and unrecognized drug/drug interactions could influence the degree of suppression of E_2_ in the individual patient. To allow optimum clinical decision making, E_2_ levels have to be measured and documented properly, requiring highly sensitive and specific assays. Our study clearly shows that E_2_ measurements based on the immunoassay methods that lack appropriate preceding purification steps do not fulfill this need. The use of incorrectly performing commercial E_2_ kits could lead to misguided clinical decisions based on inaccurate laboratory data.

The poor performance of the assays obtained with the E_2_ immunoassay kits investigated in our study can be explained by the fact that manufacturers of these kits generally do not carry out a thorough validation of the assays obtained with the kits. This is especially true regarding assay sensitivity and specificity, as evident in the present study. Assay sensitivity can be defined as the lowest concentration of a compound that can be distinguished from a sample that does not contain that compound (the zero standard). The variation of the zero standard in an immunoassay can be estimated by assaying replicates of this standard, e.g., 10 replicates, and calculating the mean counts bound and the standard deviation. The mean counts minus 2 standard deviations, read off the assay standard curve as the concentration (by extrapolation), is the minimal detection limit. However, manufacturers of steroid hormone immunoassay kits rarely state how they determine the assay sensitivity, which is reported in their package inserts describing the assay validation. Often they report a sensitivity that is lower than the lowest point on the standard curve, which they obtain by extrapolation. This is evident in Table 1, which shows lower values reported by manufacturers of the Siemens Coat-A-Count Estradiol RIA, Siemens Double Antibody RIA and Beckman Couter Ultra-Sensitive RIA. Values measured below the lowest reliable point on the standard curve should be reported as being below assay sensitivity. Since in the present study, the lowest E_2_ concentration obtained in the standard curve was 20 pg/ml with three of the kits and 5 pg/ml with the other three kits, E_2_ levels below these concentrations would not be reliable. One of the assays in which the lowest E_2_ standard was 5 pg/ml had a sensitivity of 1.4 pg/ml according to the manufacturer of the kit (Siemens Double Antibody RIA kit). A total of 72 of the 76 E_2_ measurements obtained with this assay were <5 pg/ml. Although at first it appears that this assay is highly sensitive, we have previously shown that the assay underestimates the true E_2_ measurements and lacks specificity (Stanczyk et al. 2010).

Assay specificity is defined as the degree of interference or cross-reaction encountered from substances other than the one that is measured in the assay. The most obvious potential cross-reacting substances are metabolites of E_2_, which include unconjugated metabolites such as estrone (E_1_), as well as conjugated metabolites (sulfates and glucuronides) of both E_2_ and E_1_. The total number of E_2_ metabolites is over 100. In addition to potential interference in the assay by E_2_ metabolites, AIs and/or one or more of their numerous metabolites may also cross-react with the antibody in the assay. Thus, purification of E_2_ by use of organic solvent extraction to remove conjugated metabolites and chromatography to separate unconjugated metabolites from E_2_ prior to its quantitation is essential. This is not done when commercial immunoassay kits are used to measure E_2_, resulting in overestimation of E_2_ levels as observed in the present study.

## Conclusion

The findings of this study clearly emphasize the need for improved E_2_ immunoassay methods, or a complete change in methodology to ensure accurate and reliable serum E_2_ measurements for patients undergoing AI treatment. The LC-MS/MS assay is the purported gold standard for measuring steroids with high validity. Although this method is costly, cumbersome, and not widely available in every hospital setting, LC-MS/MS assays have been implemented successfully for routine steroid hormone analysis in major laboratories (e.g., Mayo Clinic, Quest Diagnostics, Esoterix and others) (Stanczyk & Clarke 2010). To ensure the correct measurement of E_2_ levels, serum samples from patients undergoing AI treatment should be sent to these specialized laboratories.

To summarize, our findings showed that commercially available and frequently used E_2_ immunoassay kits lack the sensitivity and specificity to measure the extremely low serum E_2_ levels in postmenopausal breast cancer patients undergoing AI treatment. Because most clinicians who treat such patients and send serum samples for E_2_ measurement to clinical diagnostic laboratories are not familiar with assay methodology, we would like to draw attention to this problem that could affect treatment outcome and safety of patients. Hence, the findings of this study translate into an immediate clinical need for improved immunoassay methods or a complete change in methodology so accurate and reliable serum E_2_ measurements for AI treated patients can be ensured.

### Ethics

As a requirement of publication authors have provided to the publisher signed confirmation of compliance with legal and ethical obligations including but not limited to the following: authorship and contributorship, conflicts of interest, privacy and confidentiality and (where applicable) protection of human and animal research subjects.

## Disclosures

The authors have read and confirmed their agreement with the ICMJE authorship and competing interest criteria. The authors have also confirmed that this article is unique and not under consideration or published in any other publication, and that they have permission from rights holders to reproduce any copyrighted material. Any disclosures are made in this section. The external blind peer reviewers report no competing interest.

## Funding

This study was funded by Heather R Macdonald and Frank Z Stanczyk.

## Authors’ original submitted files for images

Below are the links to the authors’ original submitted files for images. Authors’ original file for figure 1

## References

[CR1] Coates AS, Keshaviah A, Thurlimann B (2007). Five years of letrozole compared with tamoxifen as initial adjuvant therapy for postmenopausal women with endocrine-responsive early breast cancer: update of study BIG 1–98. J Clin Oncol.

[CR2] Forbes JF, Cuzick J, Buzdar A, Howell A, Tobias JS, Baum M (2008). Effect of anastrozole and tamoxifen as adjuvant treatment for early-stage breast cancer: 100-month analysis of the ATAC trial. Lancet Oncol.

[CR3] Howell A, Cuzick J, Baum M (2005). Result.s of the ATAC (arimidex, tamoxifen, alone or in combination) trial after completion of 5 years’ adjuvant treatment for breast cancer. Lancet.

[CR4] Santen RJ, Demers L, Ohorodnik S, Settlage J, Langecker P, Blanchett D, Goss PE, Wang S (2007). Superiority of gas chromatography/tandem mass spectrometry assay (GC/MS/MS) for estradiol for monitoring of aromatase inhibitor therapy. Steroids.

[CR5] Stanczyk FZ, Clarke NJ (2010). Advantages and challenges of mass spectrometry assays for steroid hormones. J Steroid Biochem Mol Biol.

[CR6] Stanczyk FZ, Jurow J, Hsing AW (2010). Limitations of direct immunoassays for measuring circulating estradiol levels in postmenopausal women and men in epidemiologic studies. Cancer Epidemiol Biomark Prev.

[CR7] Stanczyk FZ, Cho MM, Endres DB, Morrison JL, Patel S, Paulson RJ (2003). Limitations of direct estradiol and testosterone immunoassay kits. Steroids.

[CR8] Amir E, Seruga B, Niraula S, Carlsson L, Ocaña A (2011). Toxicity of adjuvant endocrine therapy in postmenopausal breast cancer patients: a systematic review and meta-analysis. J Natl Cancer Inst.

[CR9] Dowsett M, Cuzick J, Ingle J (2010). Meta-analysis of breast cancer outcomes in adjuvant trials for aromatase inhibitors versus tamoxifen. J Clin Oncol.

