# Supplementary figures and images for: Deficiencies in immunoassay methods used to monitor serum Estradiol levels during aromatase inhibitor treatment in postmenopausal breast cancer patients
Source: Springerplus. 2013 Jan 11;2(1):5. doi: 10.1186/2193-1801-2-5 (PMC3599208; doi:10.1186/2193-1801-2-5)

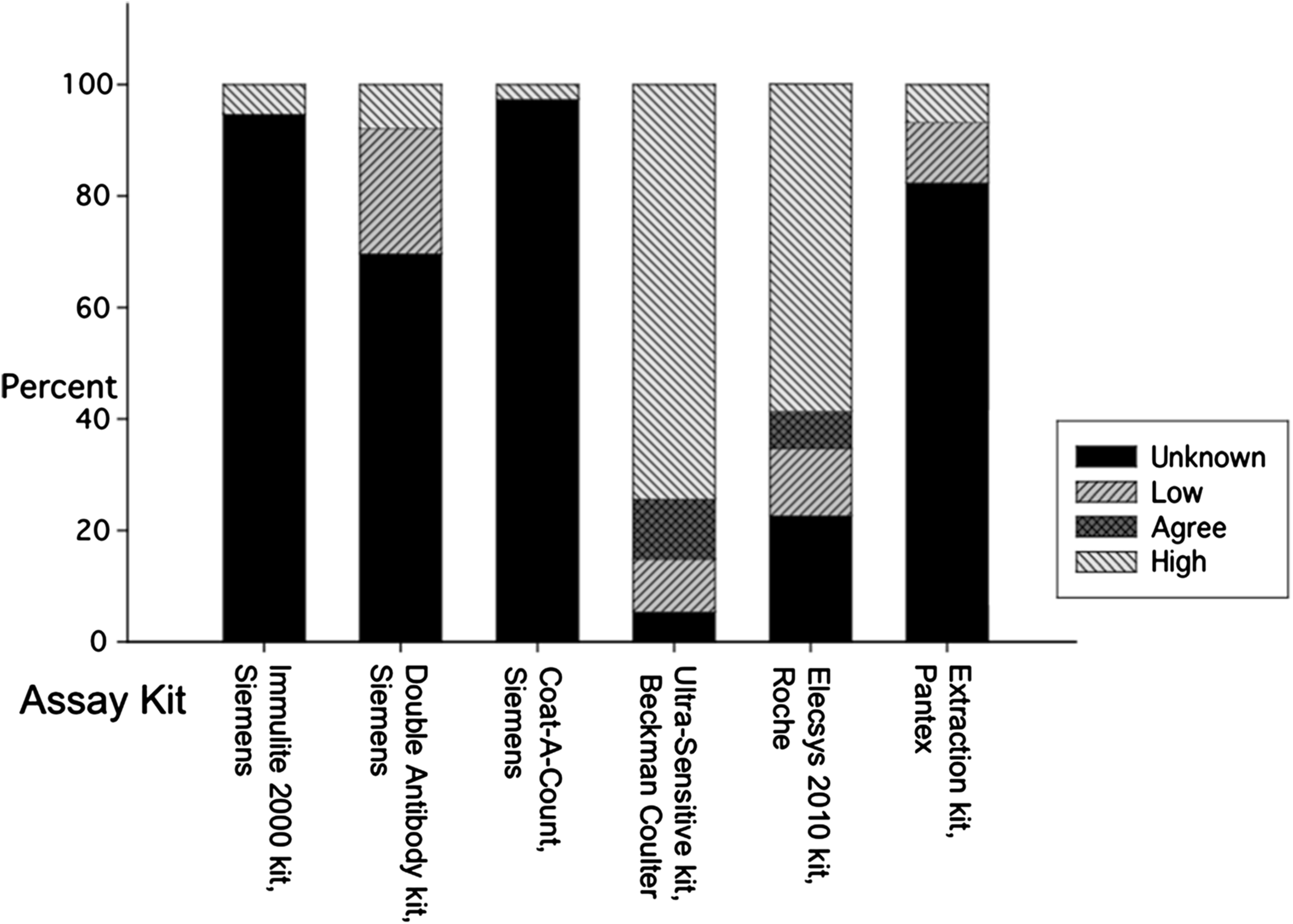

Supplement: Supplementary file 1 — Authors’ original file for figure 1 [file 40064_2012_124_MOESM1_ESM.tiff]
